# The role of conception type in the definition of primary and secondary infertility

**Published:** 2018-05

**Authors:** Firouzeh Ghaffari, Arezoo Arabipoor

**Affiliations:** *Department of Endocrinology and Female Infertility, Reproductive Biomedicine Research Center, Royan Institute for Reproductive Biomedicine, ACECR, Tehran, Iran.*

**Dear Editor**

Uniform and definitive terminology in reproductive medicine is important for appropriate timing in starting an infertility workup to avoid over- and under-treatment, their related financial burden and psychological pressures (1, 2) and comparison of different treatments. Despite surveys and definitions provided by the World Health Organization (WHO), it seems that some terms are still confusing and misleading and therefore further discussion in this area is essential (2).

The objective of the current letter is, to discuss critically a number of issues including the definitions of infertility, especially the role of assisted reproductive treatments. In the most recent definition proposed by WHO and the International Committee for Monitoring Assisted Reproductive Technology ICMART (3), clinical infertility was considered as "a disease of the reproductive system defined by the failure to achieve a clinical pregnancy after 12 months or more of regular unprotected sexual intercourse". However, it appears that in couples with sterility and no chance of spontaneous pregnancy (such as some men with azoospermia, primary hypothalamic amenorrhea, bilateral tubal ligation, some type of premature ovarian failure, etc.), this definition is illogical and the duration of the infertility should be considered equal to the time that the couple has tried to achieve a pregnancy. A broad spectrum of patients with different histories is observed among infertile couples who are referred to treatment centers. On the one hand there are couples who have not experienced any clinical pregnancy, on the other hand there are those who have a child and are trying to have another child, and there are some women who have experienced abortion and after that never have conceived again. On the basis of WHO and International Committee for Monitoring Assisted Reproductive Technology definitions (3), the role of infertility treatment, specially assisted reproduction treatment, is not considered. Achieving clinical pregnancy in couples who have had a previous spontaneous pregnancy is very different in comparison with those who have not ever conceived and this gives them a much better prognosis (4). It seems that for evaluation of the efficacy of different infertility treatments, particularly assisted reproduction treatments, it is more appropriate that the primary infertility be defined as "inability to achieve a spontaneous clinical pregnancy". By this definition, patients who achieve the clinical pregnancy by using different infertility treatments for example: medical, intrauterine insemination and assisted reproduction technology or surgery, have not achieved a natural clinical pregnancy; the same would be true of primary infertility. So, it is more appropriate that secondary infertility be defined as "the inability to achieve a spontaneous clinical pregnancy following a previous spontaneous pregnancy". However, the main question is which type of treatment should be considered in this definition; only assisted reproduction technology cycles or any infertility treatment? In all infertility treatments, the focus is to have a live child which will survive; however, in most infertility research one of the main outcomes is the capacity to achieve a clinical pregnancy. By this new definition, the efficacy of different infertility treatments for the broad spectrum of infertile patients with different obstetrical histories could be better compared with each other. Despite successful conception, several factors are involved to reach a clinical pregnancy to live birth at term. It seems that the WHO classifications are not sufficiently detailed for these situations which have been described and require revision.

The type of conception should be considered in the definition of primary and secondary infertility. Three important factors should be considered before starting an infertility workup: the most important factors are the age of the woman, followed by the time attempting pregnancy, and the cause of infertility. Immediate infertility treatment should be suggested for the couple who are sterile (e.g. azoospermia, primary hypothalamic amenorrhea and bilateral tubal ligation etc...). More time could be allowed for couples with a good prognosis (e.g. women under 35 yr old, unexplained infertility etc…) to achieve a spontaneous pregnancy.
